# The mechanical and frost resistance properties of pressed concrete blocks mixed with the polymeric aluminum chloride waste residue

**DOI:** 10.1038/s41598-024-61347-1

**Published:** 2024-05-27

**Authors:** Ping Xu, Jin Tong, Rui Shi

**Affiliations:** 1 School of Civil Engineering, Henan Polytechnic University, Jiaozuo, 454003 Henan China; 2 International Joint Research Laboratory of Henan Province for Underground Space Development and Disaster Prevention, Henan Polytechnic University, Jiaozuo, 454003 Henan China

**Keywords:** Polymeric aluminum chloride waste residue, Pressed concrete blocks, Mechanical properties, Frost resistance, Civil engineering, Structural materials

## Abstract

This study aims to research on the mechanical and frost resistance properties of pressed concrete blocks mixed with the polymeric aluminum chloride (PAC) waste residue. Experimental studies on the activity index of volcanic ash, mechanical property, frost resistance and microstructure of pressed concrete blocks mixed with PAC waste residue were carried out. The results show that the activity index of volcanic ash of PAC waste residue reaches 74.96% at a particle size of 0.075 mm or less and a curing age of 28 days. Based on results of mechanical property tests, the optimum dosage of PAC waste residue is 15%, at which time the compressive and bending strength only decreases by 14.57% and 15.84%. Based on results of frost resistance tests, the optimum dosage of PAC waste residue for pressed concrete blocks is 10%. After 50 freeze–thaw cycles, when the dosage of PAC waste residue is 10%, the strength loss rate is only 3.04%. XRD and SEM tests show that PAC waste residue participates in chemical reactions. With a small amount of PAC waste residue, the structure of the specimen remains dense and therefore the strength decreases less.

## Introduction

The polymeric aluminum chloride (PAC) is a kind of water purifier, inorganic polymer coagulant. It has strong bridging and adsorption properties, and in the process of hydrolysis, it is accompanied by physicochemical processes such as coagulation, adsorption and precipitation. It is famous for its fast settling speed, strong adaptability to water temperature and excellent performance^1–3^. PAC is mainly used for urban water supply and drainage purification, industrial water purification, etc. It is currently internationally recognized as an excellent water purifier^4,5^. PAC is produced in large quantities due to its excellent performance. However, the common method of producing PAC is the two-step method of acid dissolution of bauxite and calcium aluminate powders. Bauxite and calcium aluminate powder are reacted with hydrochloric acid or mixed acid under certain conditions to obtain liquid PAC and solid waste residue (PAC waste residue). The amount of PAC waste residue increases with the growing demand for water purification. Typically, this waste residue is treated with simple methods^6,7^ and disposed of in landfills, posing significant pollution risks to groundwater, soil, and the atmosphere. This situation also hampers the development of the water purification industry. To mitigate pollution, scholars have proposed the concept of “used into treasure” by utilizing PAC waste residue^8,9^. Addressing the pollution issues associated with PAC waste residue also helps reduce the need for cement.

Neutral modification of PAC waste residue is a prerequisite for resource utilization. The waste residue can be treated by adding alkaline substances, mixing with water, and thoroughly stirring, followed by water removal and drying. This method effectively eliminates chlorides and acidity in PAC waste residue. Li^6^ demonstrated waste residue neutralization by adding lime, resulting in a chemical composition of PAC waste residue after chloride removal, primarily consisting of SiO_2_, Al_2_O_3_, and CaO, etc. As a byproduct of industrial PAC production, PAC waste residue is currently an area of focus for recycling and reuse in the chemical field. For instance, Li^6^ employed quicklime to modify and neutralize PAC waste residue to produce a decolorizing agent. In general, the volcanic ash activity of volcanic gray materials is directly proportional to the content of amorphous silicate and aluminum acid^10–12^. Since PAC waste residue contains significant amount of SiO_2_ and Al_2_O_3_, it can partially replace cement to manufacture concrete^13–16^. The particle size of industrial waste sludge significantly influences the volcanic activity index^17–20^. Li^21^ depicts that reducing particle size can effectively improve the red mud volcanic activity index. Therefore, this paper improve the volcanic activity index of PAC waste residue through crushing, thereby maximizing its utilization.

In this paper, according to the volcanic activity index as well as granular characteristics of PAC waste residue, PAC waste residue was used to replace part of the cement, and method of press molding using hydrostatic driving force was used to prepare concrete pavement bricks with a large amount of market usage, call it pressed concrete blocks. The bricks produced by this process do not need to be sintered, as long as the natural maintenance or autoclave maintenance can be avoided, avoiding the pollution caused by high temperature combustion of waste residue, but also to prevent the change of the nature of the waste residue. And the dry apparent density, water absorption, compressive strength, flexural strength and frost resistance were used as the main technical indexes for evaluating the performance of the pavement bricks.

## Test preparation

### Materials

**Figure 1:**
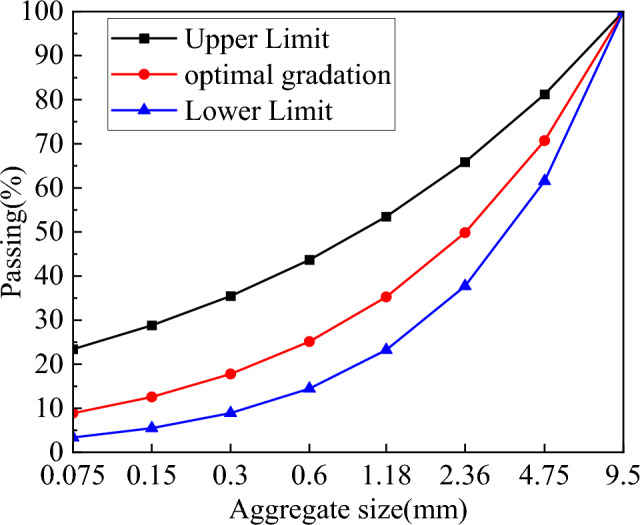
Aggregate grading curve.

The experiment employs P.O 42.5 ordinary silicate cement as the primary material. Table 1 presents its chemical composition. The fine aggregate used is a standard sand with a density of 2.58 g/cm^3^ and a fine degree of granularity. The coarse aggregate was natural gravel, maximum particle size is 9.5 mm. Its apparent density measures 2652 kg/m^3^, and the crushing value is 6.9%. Using Fuller grading curves for aggregate grading design, a synthetic grading with 70% mechanism sand and 30% gravel is shown in Fig. 1.

**Table 1:** Chemical composition of cement and PAC waste residue (%).

	SiO_2_	Al_2_O_3_	Fe_2_O_3_	CaO	MgO	TiO_2_	Cl^−^	Others
Cement	20.15	4.76	3.18	64.87	2.87	–	–	1.74
PAC waste residue	45.72	27.80	3.42	5.68	1.34	3.86	2.25	10.39

**Figure 2:**
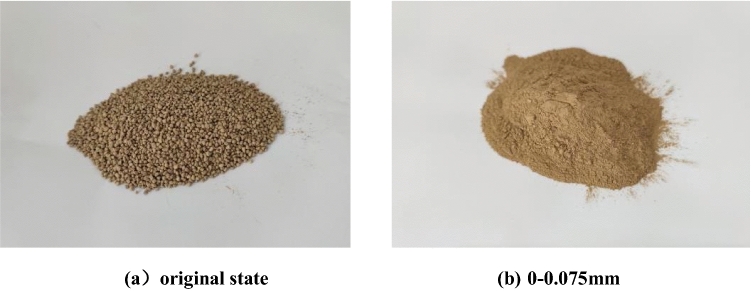
Appearance of PAC waste residue. (**a**) Original state. (**b**) 0–0.075 mm.

The PAC waste residue is derived from the waste produced during the industrial production of water purification agents. It possesses a mild acidic nature and cannot be directly utilized as a cement admixture. Consequently, it necessitates dechlorination. The dechlorination process for PAC waste residue involves the following steps: first, the PAC waste residue is mixed with quicklime, ensuring a thorough stirring. Second, the water is added to prepare a slurry, which is then mixed and allowed to stand until the slurry naturally separates into supernatant liquid and waste slag precipitates. Third, the supernatant is removed, leaving behind the waste residue sediment. Finally, the waste residue sediment is calcined and dried at a low temperature of 150 ℃–200 ℃. The supernatant obtained after standing is rich in CaCl_2_. The removal of the supernatant effectively eliminates chloride ions and acids. The chlorinated PAC waste residue with a particle size of 0.075 mm is prepared through the processes of crushing, and sieving. The original state of the PAC waste residue is powdery particles bonded into blocks, the crushing process only need to use a rubber hammer to repeatedly hammer, the block of PAC waste residue crushed into powder, screened with a screen can be obtained particle size less than 0.075 mm PAC waste residue. The appearance of PAC waste residue is shown in Fig. 2.

**Figure 3:**
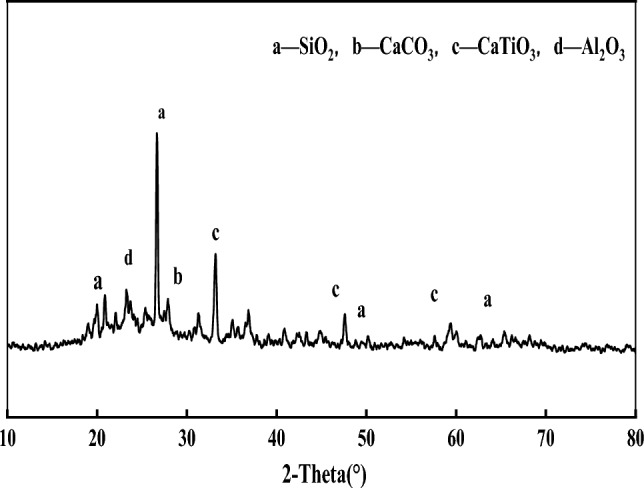
XRD pattern of PAC waste residue.

**Figure 4:**
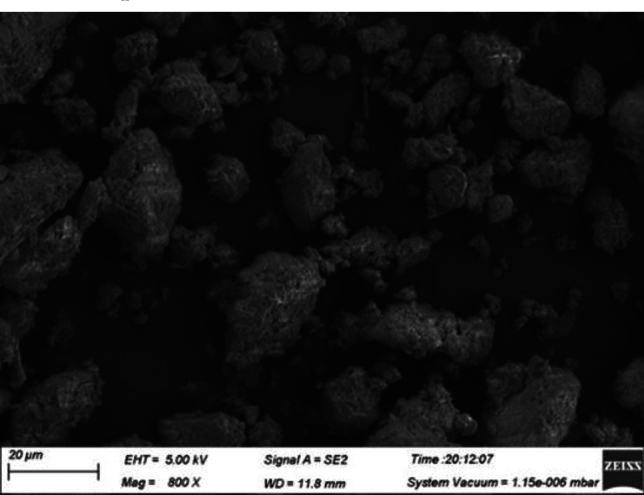
Microscopic morphology of PAC waste residue.

Table 1 provides the chemical composition of the PAC waste residue, while Figs. 3 and 4 depict the XRD pattern and microscopic morphology, respectively. As can be seen from Fig. 3 and Table 1, the dechlorinated PAC waste residue contains more crystalline phases such as SiO_2_, Al_2_O_3_, CaCO_3_ and CaTiO_3_ crystals. Among them, SiO_2_ accounts for 45.72% of the chemical composition, and Al_2_O_3_ accounts for 27.80% of the chemical composition. Therefore, PAC waste residue is suitable as a concrete admixture. As can be seen from Fig. 4, PAC waste residue particles are very unevenly distributed and have a rough apparent morphology. This will increase the friction between the particles, which will cause a decrease in the fluidity of the concrete and affect the ease of concrete. Therefore, a higher water-to-cement ratio is required.

### Test model preparation and test method

The proportion of materials used in the pressed concrete blocks formulation consists of 17% cement, 58% fine aggregate, and 25% coarse aggregate. Water to cement ratio was 0.40. Six groups of mix proportion were set in this experiment, which were P0, P10, P15, P20, P25, and P30, respectively. Table 2 provides the mix proportion of each specimen.

**Table 2:** Mix proportion of specimen.

Codes	Amount of material per unit volume (kg/m^3^)				
	Cement	Water	Coarse aggregate	Fine aggregate	PAC waste
P0	360	144	523.8	1222.2	0
P10	324	144	523.8	1222.2	36
P15	306	144	523.8	1222.2	54
P20	288	144	523.8	1222.2	72
P25	270	144	523.8	1222.2	90
P30	252	144	523.8	1222.2	108

The process of preparing pressed concrete blocks involves the following steps: thoroughly mixing the cement and PAC waste residue according to the prescribed ratio. Adding the coarse aggregate to the horizontal mixer and stirring it well. Incorporating a specific amount of water into the mixture and continuing to stir. Pouring the mixture into molds with dimensions of 200 mm × 100 mm × 60 mm. Use ZY500 automatic hydrostatic brick machine to slowly apply pressure to the mixture. The molding pressure is 5 MPa. Maintaining the pressure for a certain duration to create the pressed concrete blocks test specimens. Transferring the specimens to a standard a standard curing room with a temperature of 20 ± 2 ℃ and a relative humidity of 95% or more. Evaluating the performance of pressed concrete blocks once they have reached the curing age.

**Figure 5:**
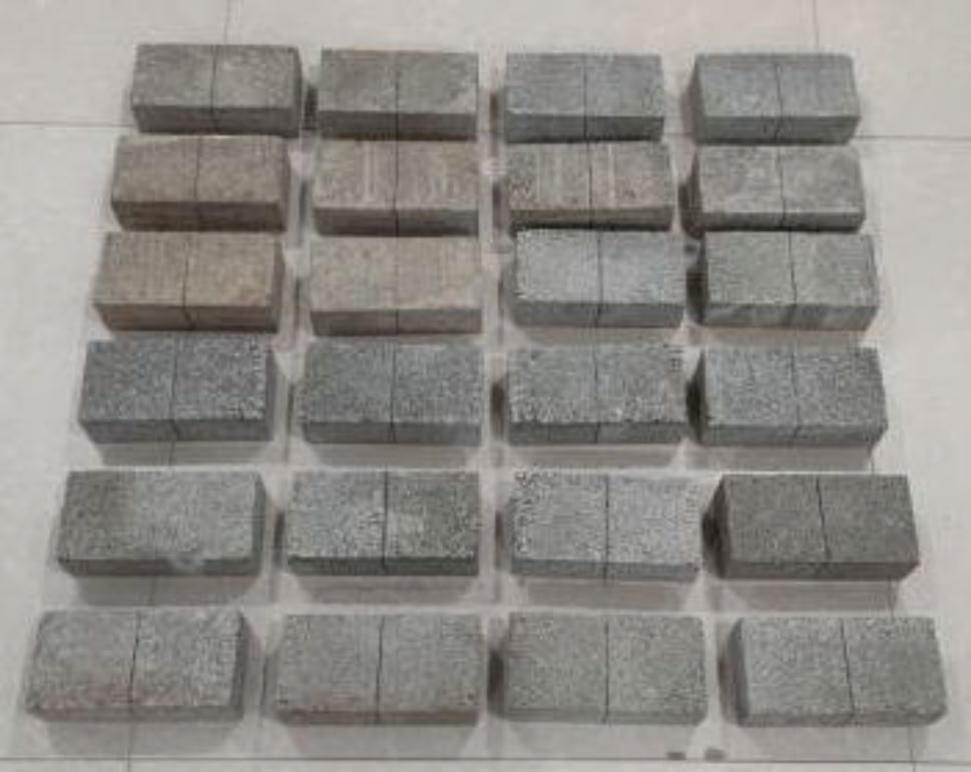
Appearance of some specimens.

The performance tests conducted on the pressed concrete blocks include assessing their volcanic activity index, dry weight density, water absorption, compressive strength, bending strength, and frost resistance. Refer to GB 28635-2012 “Concrete pavement Brick” for testing. Pressed concrete blocks specimen size is 200 mm × 100 mm × 60 mm. The 7 days compressive strength was tested for both P0 and P30 groups. Six groups of P0–P30 were tested for 28 days compressive and bending strength. Freeze–thaw tests were conducted at 25, 35 and 50 cycles for five groups of P0–P25. Three specimens were set up for each test group, and a total of 87 specimens were produced. The closest of the three specimens to the average value was selected for the test results. The appearance of some specimens is shown in Fig. 5, From the figure, it can be seen that the specimen with more PAC waste residue is yellowish in appearance, which is due to the fact that PAC waste residue is a yellow powder, while the specimen is pressed and molded, and the appearance of the specimen retains the color of the raw material.

**Figure 6:**
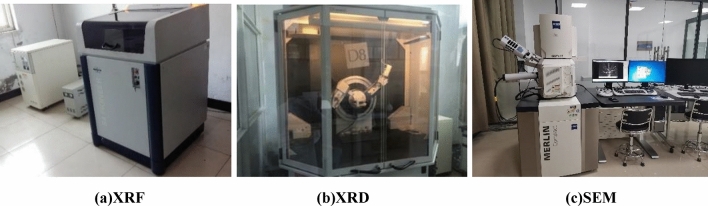
Microscopic testing instruments. (**a**) XRF. (**b**)XRD. (**c**) SEM.

In order to further analyze the mechanism of PAC waste residue in pressed concrete blocks, the chemical composition of dechlorinated PAC waste residue was determined by X-ray diffractometry (XRD) and fluorescence spectrometry (XRF). To examine the microstructure, the Merlin compact scanned electronic microscope, manufactured by Carl Zeiss NTS GmbH, is employed for electron microscopy analysis. The microscopic test instruments are shown in Fig. 6. After compression test, collection of broken pieces of crushed hydrostatic concrete block specimens. Among them, the pieces from the freeze–thaw cycle are dried in a freeze dryer for 24 h. The collected pieces were broken down to obtain fresh samples. Fresh samples were analyzed by microscopic testing using the above instruments.

## Results and discussion

### Testing of the PAC waste residue volcanic activity index

The potential of utilizing PAC waste residue as a substitute for cement in concrete primarily depends on the volcanic ash activity of the PAC waste residue. This study aims to assess the volcanic activity index of the original PAC waste residue and the PAC waste residue with a particle size below 0.07 mm.

**Figure 7:**
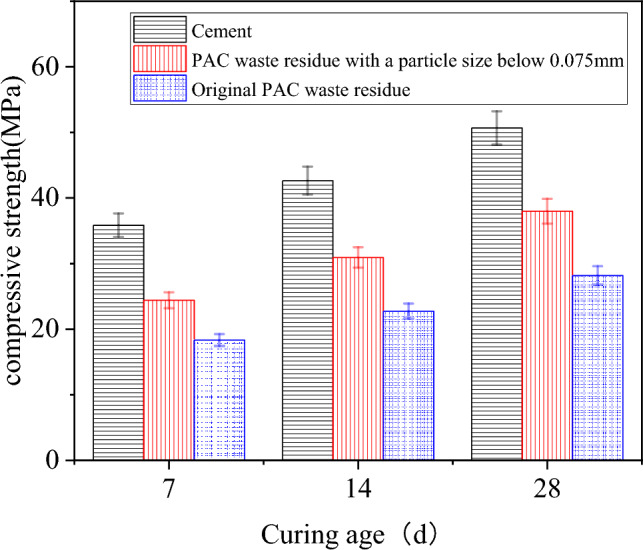
Compressive strength of the three sets of cured specimens.

To evaluate the feasibility, concrete specimens were prepared with 30% of cement replaced by PAC waste residue. The compressive strength of standard specimens was tested at curing ages of 7, 14 and 28 days, and the compressive strength ratio was calculated. The results of the compressive strength test for the three sets of cured specimens are illustrated in Fig. 7.

According to the results depicted in Fig. 7 and the volcanic activity index calculation formula (A = H_t_/H_0_ × 100%, where A is the volcanic activity index, H_t_ represents the compressive strength of the PAC waste residue cement mortar, and H_0_ denotes the compressive strength of the benchmark group). The volcanic activity indexes for 7, 14, and 28 days of the original PAC waste residue were determined as 51.13%, 53.34%, and 55.62%, respectively. The volcanic activity indexes for 7, 14, and 28 days of the PAC waste residue with a particle size below 0.075 mm also were determined as 68.06%, 72.52%, and 74.96%, respectively. These results indicate a significant improvement in the volcanic activity index by crushing the PAC waste residue particles. The 7 day and 28 day volcanic activity indexes should exceed 65% and 70% when using PAC waste residue as a blend in concrete products^22^. The original PAC waste residue exhibits low volcanic activity indexes at 7 days and 28 days, rendering it unsuitable for direct use. However, the volcanic activity index increases by reducing the particle size of the PAC waste residue through crushing. The volcanic activity index of PAC waste residue with a particle size below 0.075 mm is more than 65% and 70%. This proves that PAC waste residue with particle size less than 0.075 mm meets the requirements as concrete admixture and has the potential to replace cement. Therefore, PAC waste residue with less than 0.075 mm particle sizes is selected for further testing.

**Figure 8:**
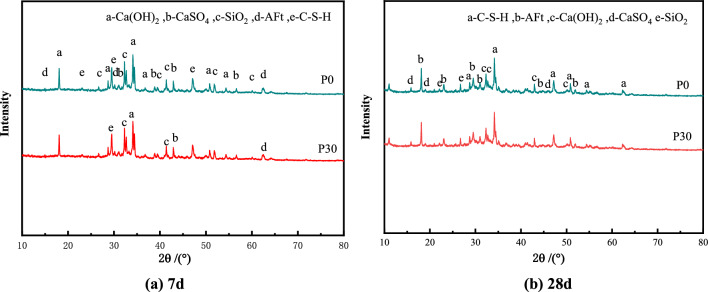
XRD analysis results of P0 group and P30 group specimens. (**a**) 7 days. (**b**) 28 days.

**Figure 9:**
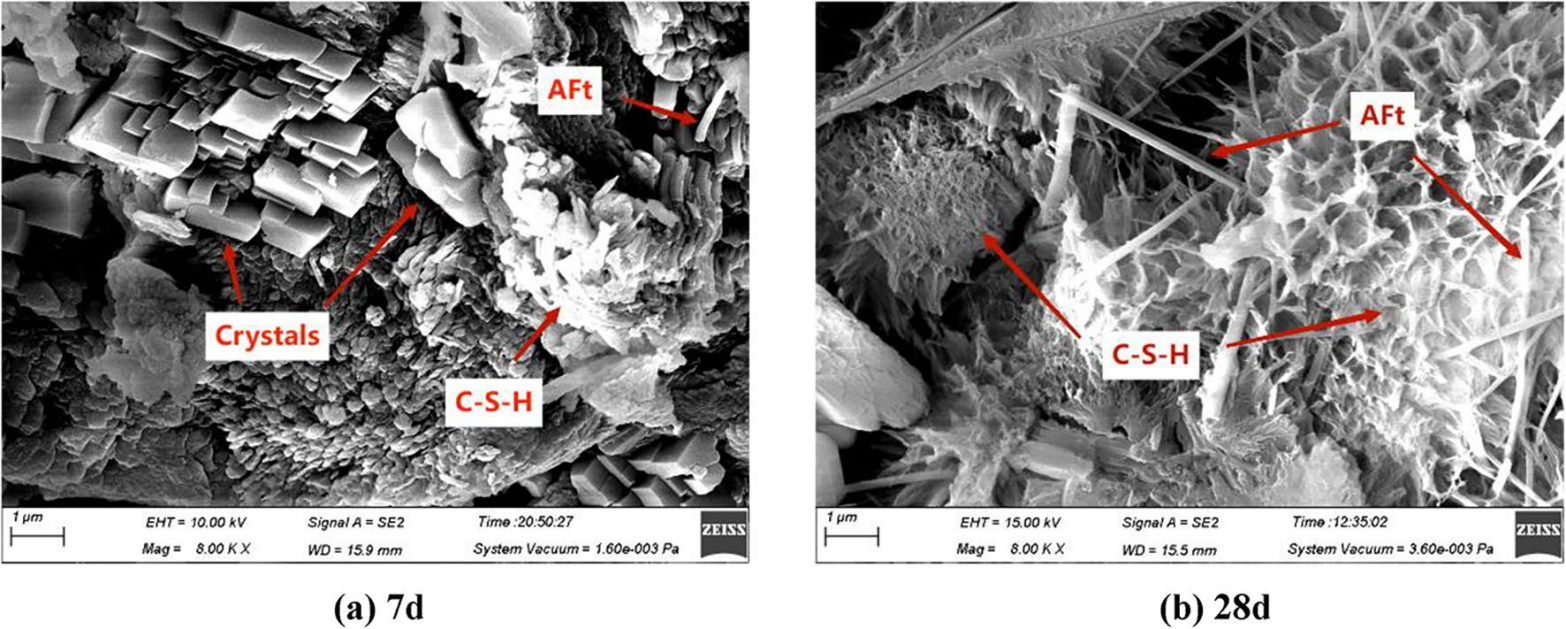
SEM testing of P30 group specimens. (**a**) 7 days. (**b**) 28 days.

To further analyze the feasibility of replacing cement with PAC waste residue in pressed concrete blocks, tests were conducted using concrete specimens of P0 and P30 groups. Figure 8 presents the XRD maps of the P0 group and P30 group specimens at 7 days and 28 days, while Fig. 9 displays the SEM diagram of the P30 group sample at 7 days and 28 days.

Analyzing the results from Figs. 8 and 9, it can be observed that the P0 group of specimens mainly consist of Ca(OH)_2_ crystals, a small amount of ettringite (Aft) and calcium-silicate-hydrates (C–S–H) gels. Additionally, a small amount of calcium carbonate generated through natural carbonization is present. The diffraction peaks of SiO_2_ mixed with the dechlorinated PAC waste residue sample are observed at 28 days. No new crystal formation occurs in the concrete specimens with de-chlorinated PAC waste residue. P30 group concrete specimens compared to P0 group concrete specimens, the main peak of Ca(OH)_2_ and characteristic peaks of and characteristic peaks of calcium-silicate-hydrates (C–S–H) gels in the hydrophilic products at 7 days are not significantly different. However, over time, the volcanic ash reaction of the de-chlorinated PAC waste residue consumes a portion of Ca(OH)_2_.This is evident from weakened main peaks of Ca(OH)_2_ and calcium-silicate-hydrates (C–S–H) trigly in the hydrophilic products. The test results indicate PAC waste residue reaction with cement hydrated products, gradually reducing the Ca(OH)_2_ content in the cement hydrated products. The reason for this phenomenon is that CaO in cement reacts with water to form Ca(OH)_2_, dissolved SiO_2_ and Al_2_O_3_ in PAC waste residue react with Ca(OH)_2_ to form hydrated calcium silicate(C–S–H) and hydrated calcium aluminate(C–A–H) gels, hydrated calcium aluminate(C–A–H) reacts with CaSO_4_ to produce ettringite (Aft). The reaction mechanism is as follows:

xCa(OH)_2_ + SiO_2_ + mH_2_O → xCaO-SiO_2_-nH_2_O

xCa(OH)_2_ + Al_2_O_3_ + mH_2_O → xCaO-Al_2_O_3_-nH_2_O

C_3_A + 3(CaSO_4_·2H_2_O) + 2Ca(OH)_2_ + 24H_2_O → 3CaO·Al_2_O_3_·3CaSO_4_·32H_2_O

Figure 9 clearly demonstrates that as age increases, the hydration product of the P30 group Concrete specimens significantly increases. Notably, there is a noticeable increase in the calcium-silicate-hydrates (C–S–H) and ettringite (Aft) between pores. These observations indicate that as the hydration reaction proceeds, the activity of dechlorinated PAC waste residue is gradually released after 7 days of aging, leading to a volcanic ash reaction that promotes the production of hydrophilic products.

### Effect of different dosage of PAC waste residue on the mechanical properties of pressed concrete blocks

**Figure 10:**
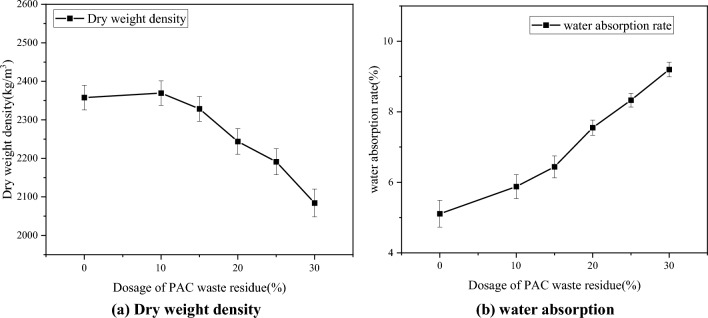
Dry weight density and water absorption. (**a**) Dry weight density. (**b**) Water absorption.

**Figure 11:**
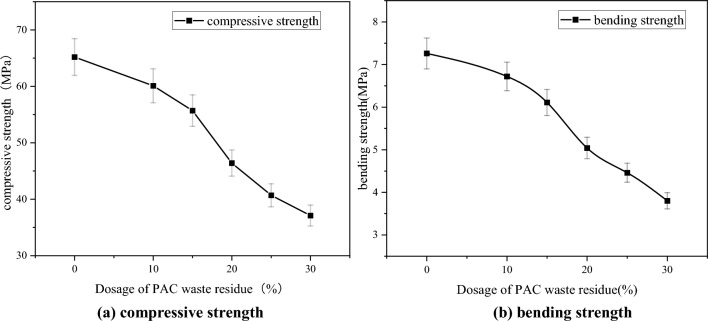
Compressive strength and bending strength. (**a**) Compressive strength. (**b**) Bending strength.

This experiment aimed to investigate the impact of replacing cement with PAC waste residue on the properties of pressed concrete blocks, including dry weight density, water absorption, compressive strength, and bending strength. The test results, obtained from the specimens after 28 days of curing, are presented in Figs. 10 and 11.

Figure 10 reveals that when PAC waste residue replaces cement, there is a decrease in the dry weight density of pressed concrete blocks, accompanied by an increase in water absorption rate. When the PAC waste residue replaces 15% of the cement, the dry weight density of pressed concrete blocks is reduced by 4.88%, while the water absorption rate increases by 26.03%. When the PAC waste residue replaces 30% of the cement, the dry weight density of pressed concrete blocks is reduced by 11.61%, while the water absorption rate increases by 80.07%. Figure 11 demonstrates that as the amount of PAC waste residue replacing cement increases, the pressed concrete blocks’s compressive strength and bending strength. When the PAC waste residue replaces 15% of the cement, the compressive strength of pressed concrete blocks is reduced by 14.57%, while the bending strength decreases by 15.84%. When the PAC waste residue replaces 30% of the cement, the compressive strength of pressed concrete blocks is reduced by 36.96%, while the bending strength decreases by 47.65%. These results indicate a significant decline in the mechanical properties of pressed concrete blocks. The decrease in strength is less when the PAC waste residue is below 15%. After exceeding 15%, the decrease increases with the increase of dosage. In view of the above, before and after the PAC waste residue dosing of 15%, there is a significant difference in the trend of each property change. PAC waste residue exhibits potential hydration activity. By incorporating an appropriate amount of PAC waste residue, the pore structure of the pressed concrete blocks can be improved. The generated Ca(OH)_2_ from cement hydration and the calcium–silicate–hydrates (C–S–H) can fill the concrete pores, compensating for the lower activity of PAC waste residue. However, exceeding the optimal value of the dosage of PAC waste residue can lead to insufficient secondary hydration, increased micro-crack defects in hydration products, and elevated water demand for the hydration reaction, thus reducing the strength. Combined with the above test results, the optimal value of PAC waste residue dosage is 15%.

### Effect of different dosage of PAC waste residue on the frost resistance of pressed concrete blocks

**Figure 12:**
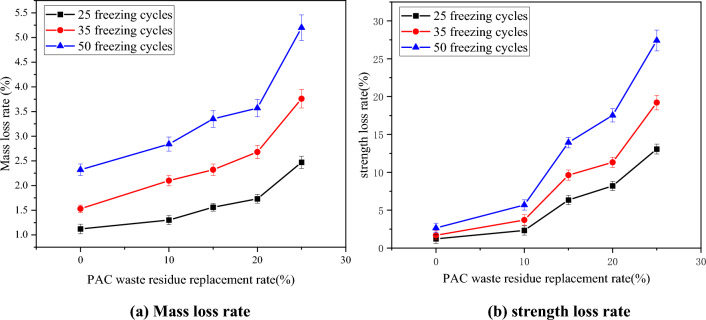
Frost resistance of pressed concrete blocks. (**a**) Mass loss rate. (**b**) Strength loss rate.

The test results of PAC waste residue dosage on the frost resistance of concrete hydrostatic bricks are shown in Fig. 12.

Figure 12a illustrates that during the initial freezing stage, the damage caused by freezing to the specimens of pressed concrete blocks was relatively minor, and the loss in mass was not significantly different between the various doping conditions. However, as the freezing cycles progressed, the freezing and thawing process deepened the damage to the tested parts, resulting in a significant increase in mass loss. This can be attributed to the PAC waste residue, which led to a higher water absorption rate. The effects became more evident prolonged freezing and thawing cycles. After 50 freezing–thawing cycles, the concrete test parts’ surface suffered severe damage, leading to a sudden increase in the mass loss rate of pressed concrete blocks test parts in each group. The higher the amount of PAC waste residue, the greater the water absorption rate of the pressed concrete blocks, resulting in more sample peeling and a higher increase in mass loss rates.

As the number of freezing–thawing cycles increased, the damage inflicted by freezing on the test parts deepened, and the impact of PAC waste residue doping on strength loss became more obvious. Figure 12b demonstrates that the resistance to freezing–thawing cycles of the pressed concrete blocks was significantly affected. This can be attributed to the strong water absorption by waste residue through hydrophilic reactions. The absorbed water does not participate in the hydration process, leading to a loss in sample strength. During the freezing–thawing cycles, the absorbed water undergoes freezing and expansion, exerting pressure on the internal structure and causing damage. Additionally, a higher internal water content generates greater freezing pressure, intensifying the damage. Therefore, as the amount of waste residue increases, the sample experiences greater strength loss. It is noteworthy that when the replacement rate exceeds 10%, the frost resistance performance declines sharply. When 10% of cement was replaced with PAC waste residue, the rate of strength loss of pressed concrete blocks in 25, 35 and 50 freeze–thaw cycles were 1.12%, 2.03% and 3.04% higher than that of the benchmark group, respectively. When 15% of cement was replaced with PAC waste residue, the rate of strength loss of pressed concrete blocks in 25, 35 and 50 freeze–thaw cycles were 5.14%, 7.95% and 11.29% higher than that of the benchmark group, respectively. Therefore, under freeze–thaw cycle conditions, it is recommended to consider the replacement rate less than 10% to optimize the use of PAC waste residue.

**Figure 13:**
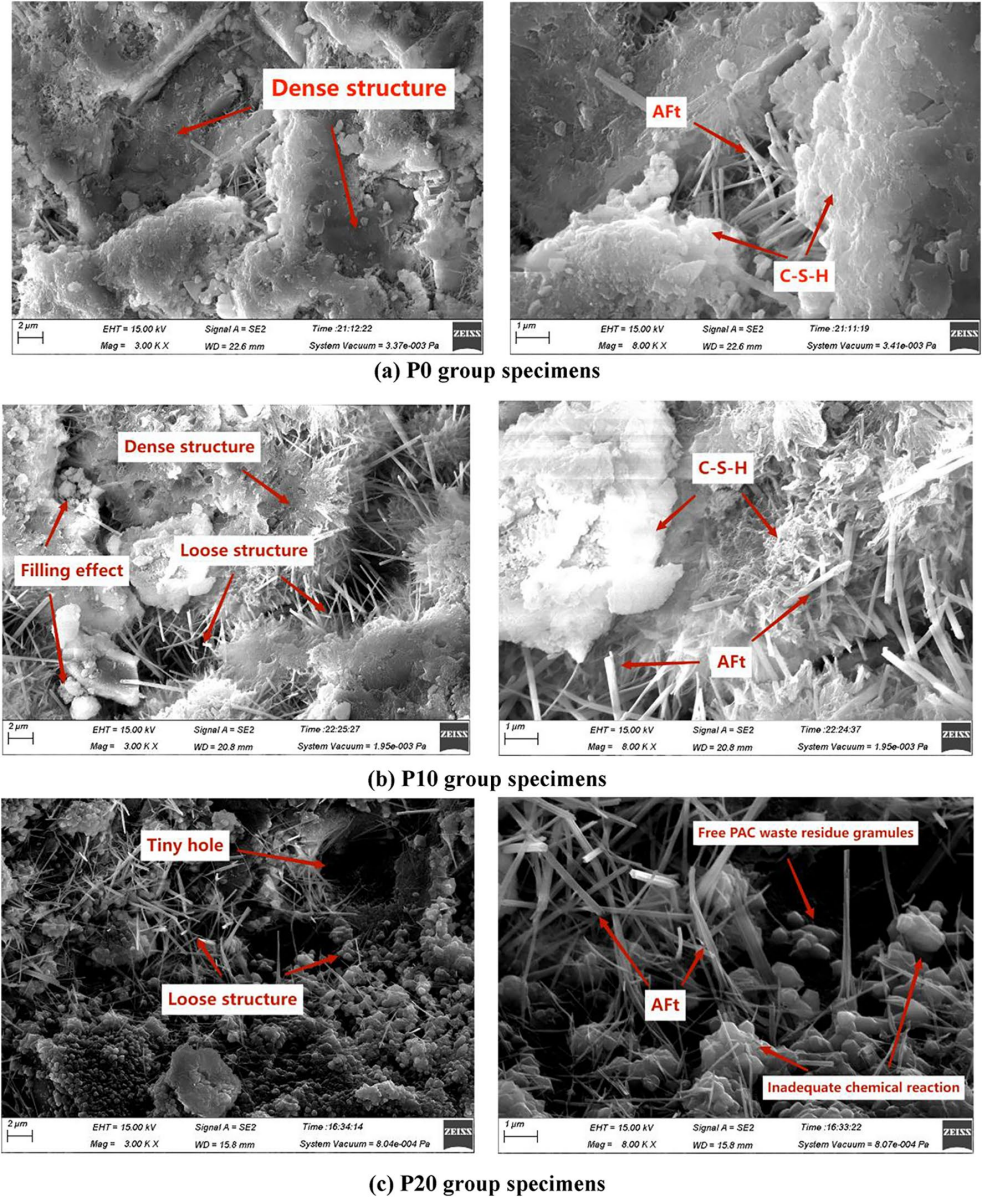
SEM testing of pressed concrete blocks. (**a**) P0 group specimens. (**b**) P10 group specimens. (**c**) P20 group specimens.

In order to reveal the mechanism of PAC waste residue in pressed concrete blocks, analyzing the microstructure of three sets of hydrostatically pressed concrete block specimens, P0, P10 and P20. Figure 13 shows the test results of three groups of specimens magnified 400 times and 2000 times, respectively.

Figure 13a reveals the presence of numerous “mesh” calcium-silicate-hydrates (C–S–H) gels and “needle-shaped” ettringite (Aft) in the test parts. The calcium–silicate–hydrates (C–S–H) gel encapsulates the ettringite (Aft), the hydration products lap each other to form a dense skeleton. The overall structure appears to be relatively dense, but there are many pores. Figure 13b demonstrates that when the doping amount of PAC waste residue is 10%, ettringite (Aft) is reduced in the specimen. The reduction in ettringite (Aft) will result in a weakening of the skeletal support of the structure and the strength will be reduced. The reason for the decrease of calcium alumina is that on the one hand, Ca^2+^ and SO_4_^2−^ decrease due to the replacement of cement. On the other hand, the large amount of Al_2_O_3_ contained in the PAC waste slag, which slowly dissolves to produce AlO^2−^ ions during the age of 28 days, and the increase in the concentration of AlO^2−^ promotes the transformation of ettringite (Aft) as follows:

C_3_A + 3CaO·Al_2_O_3_·3CaSO_4_·(30–32)H_2_O + H_2_O → 3CaO·Al_2_O_3_·CaSO4·(30–32)H_2_O(AFm)

Small substitution of cement by PAC waste results in a reduction of hydration products, but the microaggregate effect of the PAC particles allows the microstructure to remain dense, pores is reduced. This indicates that the substitution of cement with small amount of PAC waste residue does not cause a significant reduction in cement hydration products, and the microstructure remains relatively dense compared to the reference group. This is because the adopted PAC waste residue is below the particle size of 0.075 mm, enabling it to fill the pores in the transition area and the cement matrix, thus optimizing the overall pore structure of the system. The Al_2_O_3_ and SiO_2_ present in the PAC waste residue can undergo hydraulic reactions with Ca(OH)_2_, resulting in the formation of gel substances. Therefore, when the doping amount of PAC waste residue is 10%, the mechanical properties and frost resistance performance remain satisfactory without significant deterioration. However, Fig. 13c indicates that when the PAC waste residue is doped at 20%, the quantity of ettringite (Aft) significantly decreases. When the dosage of PAC waste residue is large, On the one hand, a large amount of CaO in cement undergoes a hydration reaction to form Ca(OH)_2_, the reduction of cement dosage, decrease in Ca(OH)_2_ from hydration, and the alkali content in the cement paste is reduced; On the other hand large dosage of PAC waste residue, PAC waste residue dissolved SiO_2_, Al_2_O_3_ increased. Weakly alkaline slurry will limit the full participation of the PAC waste residue in the reaction. There are free PAC waste residue particles that do not participate in the reaction, resulting in more inert components in the cementitious material. In the case of the same water consumption, it is equivalent to increasing the water–cement ratio. Therefore, although the free PAC slag particles can play a certain role in filling, due to the actual water-cement ratio is too large, the porosity of the paste structure is very large, resulting in a reduction in the compressive strength of concrete.

## Conclusion

- (1) The particle size and curing age affect the volcanic activity index of PAC waste residue. The volcanic activity indexes for 7, 14, and 28 days of the original PAC waste residue were determined as 51.13%, 53.34%, and 55.62%, respectively. The volcanic activity indexes for 7, 14, and 28 days of the PAC waste residue with a particle size below 0.075 mm also were determined as 68.06%, 72.52%, and 74.96%, respectively. By crushing the PAC waste residue to less than 0.075 mm, the volcanic activity will be enhanced.
- (2) The compressive strength and bending strength of pressed concrete blocks decrease with the increase of PAC waste residue. Based on results of mechanical performance tests, the optimum dosage of PAC waste residue was 15%, at which time the compressive strength and bending strength only decreased by 14.57% and 15.84%.
- (3) After freeze–thaw cycles, the rate of mass loss and strength loss of pressed concrete blocks increased with the increase in the dosage of PAC waste residue. Based on results of frost resistance tests, the optimum dosage of PAC waste residue is 10%. After 50 freeze–thaw cycles, when the dosage of PAC waste residue is 10%, the strength loss rate is only 3.04%. However, when the PAC waste residue dosage is 15%, the strength loss rate is as high as 11.29%.
- (4) The results of XRD and SEM tests showed that the PAC waste residue was involved in the chemical reaction. When a small amount of PAC waste was used, the structure of the specimen was still dense and the strength reduction was less. However, with the increase of the amount, part of the PAC waste residue did not get sufficient chemical reaction and existed in free state, which caused a significant decrease in strength.

## Data Availability

Some or all data, models, or codes generated or used during the study are available from the corresponding authors by request.
